# Apolipoprotein E4 impairs spontaneous blood brain barrier repair following traumatic brain injury

**DOI:** 10.1186/s13024-018-0249-5

**Published:** 2018-04-04

**Authors:** Bevan S. Main, Sonia Villapol, Stephanie S. Sloley, David J. Barton, Maia Parsadanian, Chinyere Agbaegbu, Kathryn Stefos, Mondona S. McCann, Patricia M. Washington, Olga C. Rodriguez, Mark P. Burns

**Affiliations:** 1 Laboratory for Brain Injury and Dementia, Department of Neuroscience, Georgetown University Medical Center, Washington, DC 20057 USA; 2 Lombardi Comprehensive Cancer Center, Department of Oncology, Georgetown University Medical Center, Washington, DC 20057 USA; 3 Department of Neuroscience, Georgetown University Medical Center, New Research Building-EG11, 3970 Reservoir Rd, NW, Washington, D.C 20057 USA

**Keywords:** Apolipoprotein E, Blood brain barrier, Neurovascular unit, Pericyte, MMP-9, CD31, Lectin, TBI

## Abstract

**Background:**

Traumatic Brain Injury (TBI) is a major cause of disability and mortality, to which there is currently no comprehensive treatment. Blood Brain Barrier (BBB) dysfunction is well documented in human TBI patients, yet the molecular mechanisms that underlie this neurovascular unit (NVU) pathology remains unclear. The apolipoprotein-E (apoE) protein has been implicated in controlling BBB integrity in an isoform dependent manner, via suppression of Cyclophilin A (CypA)–Matrix metallopeptidase-9 (MMP-9) signaling cascades, however the contribution of this pathway in TBI-induced BBB permeability is not fully investigated.

**Methods:**

We exposed C57Bl/6 mice to controlled cortical impact and assessed NVU and BBB permeability responses up to 21 days post-injury. We pharmacologically probed the role of the CypA-MMP-9 pathway in BBB permeability after TBI using Cyclosporin A (CsA, 20 mg/kg). Finally, as the apoE4 protein is known to be functionally deficient compared to the apoE3 protein, we used humanized APOE mice as a clinically relevant model to study the role of apoE on BBB injury and repair after TBI.

**Results:**

In C57Bl/6 mice there was an inverse relationship between soluble apoE and BBB permeability, such that damaged BBB stabilizes as apoE levels increase in the days following TBI. TBI mice displayed acute pericyte loss, increased MMP-9 production and activity, and reduced tight-junction expression. Treatment with the CypA antagonist CsA in C57Bl/6 mice attenuates MMP-9 responses and enhances BBB repair after injury, demonstrating that MMP-9 plays an important role in the timing of spontaneous BBB repair after TBI. We also show that *apoe* mRNA is present in both astrocytes and pericytes after TBI.

We report that APOE3 and APOE4 mice have similar acute BBB responses to TBI, but APOE3 mice display faster spontaneous BBB repair than APOE4 mice. Isolated microvessel analysis reveals delayed pericyte repopulation, augmented and sustained MMP-9 expression at the NVU, and impaired stabilization of Zonula Occludens-1, Occludin and Claudin-5 expression at tight junctions in APOE4 mice after TBI compared to APOE3 mice.

**Conclusions:**

These data confirm apoE as an important modulator of spontaneous BBB stabilization following TBI, and highlights the APOE4 allele as a risk factor for poor outcome after TBI.

## Background

Traumatic Brain Injury (TBI) is defined as a head injury sustained from an external physical force, resulting in the disruption of normal brain function. It represents a significant socio-economic and public health burden, with an estimated 2.5 million Americans sustaining a TBI per year, placing an extremely high economic cost on the community, currently estimated at $76.5 billion annually. It is well established that changes in blood brain barrier (BBB) function are a major determinant of TBI severity and recovery timeframes [1]. In addition to primary damage resulting from the initial impact, alterations in BBB integrity can also be influenced by deleterious secondary injury responses, including inflammatory cascades and abnormal metabolic processes within the central nervous system [2].

The human apolipoprotein-E protein (apoE) is a protein produced primarily by astrocytes and serves as a major lipid transport molecule in the central nervous system [3]. ApoE deficient mice have greater cell death, axonal pathology, and behavioral deficits after brain injury [4, 5], and apoE infusion reduces neuronal cell death in experimental ischemia [6]. Severe human TBI patients show a 70% decrease in cerebrospinal fluid apoE acutely post-injury [7], and we see a similar acute decrease in soluble apoE protein levels in mouse TBI brain [8]. Critically, the apoE protein regulates BBB permeability by regulating the activity of an LRP1-Cyclophilin A (CypA)–Matrix Metallopeptidase-9 (MMP-9) signaling pathway in pericytes located at the neurovascular unit (NVU) [9].

The human apolipoprotein-E gene (APOE) and its 3 associated polymorphisms (APOE2, APOE3, and APOE4) are hypothesized to contribute to several secondary injury processes [8], including influencing BBB breakdown [10, 11]. Clinically, the APOE4 gene polymorphism is associated with unfavorable outcomes following TBI [12], with the presence of at least one E4 allele associated with increased mortality [13], prolonged coma [14], poor prognosis [15], and an enhanced risk of late-onset Alzheimer’s disease [16]. These features are recapitulated in animal models of TBI, with APOE4 mice displaying increased mortality [17], impaired cognition [18], and exacerbated secondary injury pathways including amyloid beta accumulation [19] and neuroinflammation [20] compared to APOE3 mice. In non-injured APOE mice, APOE4 mice have enhanced BBB permeability compared to APOE3 and APOE2 mice [9]. Additionally, human APOE4 carriers exhibit increased cerebral spinal fluid MMP-9 expression, correlating with enhanced BBB dysfunction [21], while APOE4 post-mortem AD brains have increased CypA and MMP-9 in cortical pericytes [22]. This suggests that apoE isoforms may play a key role in regulating BBB integrity; however, the contribution of respective apoE isoforms to TBI-induced BBB permeability has not been investigated.

We hypothesized that apoE influences BBB permeability and subsequent stabilization following TBI. To test this hypothesis, we used multiple techniques to demonstrate the normal timecourse of BBB breakdown and repair after controlled cortical impact (CCI) in mice. We examined the protein levels and activity of MMP-9 in isolated microvessels after TBI, and also probed these microvessels for mRNA expression of *Apoe*, *Mmp-9*, and tight junction proteins. As the human apoE4 protein is known to be functionally deficient compared to the apoE3 protein, we also used humanized APOE mice as a clinically relevant model to study the role of apoE on BBB injury and repair after TBI. We report an inverse relationship between soluble apoE and BBB permeability after TBI, such that BBB permeability decreases as apoE levels increase over time post-injury. Additionally, we demonstrate that APOE4 mice display enhanced MMP-9 profiles and reduced tight junction protein expression, resulting in impaired BBB stabilization following TBI compared to APOE3 mice.

## Methods

### Animals

Wildtype C57Bl/6 male mice were purchased from Jackson Laboratories (Bar Harbor, ME) and were 3–4 months old at the time of injury. Male human APOE targeted replacement mice with the human APOE3 or APOE4 inserted at the endogenous murine APOE promoter on a C57BL/6 J background were 3–4 months old at the time of injury. A limitation of the work in this study is that it only included male mice. Further research will be needed to determine if these results translate to female mice.

Animals were group housed under a 12 h light/dark cycle with ad libitum access to food and water. All mice were euthanized via carbon dioxide inhalation followed by saline perfusion. All procedures were conducted in accordance with protocols approved by the Georgetown University Animal Care and Use Committee. Experiments adhered to guidelines from the Guide for the Care and Use of Laboratory Animals, U.S. Department of Health and Human Services.

### Controlled cortical impact (CCI) mouse model of TBI

CCI was conducted as previously described [8, 19]. Briefly, surgical anesthesia was induced using 4% isoflurane with maintenance in 2%, at flow rate of 1–1.5 L/min in freely breathing oxygen. The anesthetized animal was mounted in a stereotaxic frame and the surgical site clipped and aseptically sterilized. A 10 mm midline incision was made over the skull, the skin and fascia reflected and craniotomy performed (4 mm) on the central aspect of the left parietal bone. The impounder tip of a Leica StereoOne Impactor was sterilized, positioned to the surface of the exposed dura, and set to impact the cortical surface at 5.25-m/s velocity, 1.5 mm tissue deformation as previously described [23]. Sham animals received isoflurane anesthesia, skin incision, and reflection, but no impact. After injury, the incision was closed with wound clips, anesthesia discontinued, 1 ml saline administered by intraperitoneal (i.p) injection and the mouse placed in a heated cage to maintain normothermia for a 45 min recovery period. All animals monitored carefully for 4 h after surgery and then daily.

### Pharmacological treatments

For cyclosporine A (CsA) experiments, wildtype mice were randomly assigned in a blinded manner to receive either cyclosporine A (Sigma-Aldrich, St Louis, MO) or vehicle (5% ethanol, 25% cremaphor oil, and 70% saline), at 15 min post-injury. CsA was administered i.p. at 20 mg/kg in 10 mL/kg initially, with repeat doses administered at 10 mg/kg at 10 mL/kg, i.p. at 24 h intervals. The safety and efficacy of this dosing regimen in rodents has previously been established [24].

### Magnetic resonance imaging

Magnetic resonance imaging (MRI) was performed using the 7 Tesla horizontal bore Bruker Magnetic Resonance Imager in the Lombardi Comprehensive Cancer Center’s Preclinical Imaging Research Laboratory, at Georgetown University. Animals were anesthetized using 1.5% isoflurane and 30% nitrous oxide, positioned in a custom-made mouse stereotaxic device, and monitored for temperature and respiration. Following a pre-injection scan, the mice were withdrawn from the scanner and 200 μl of the gadolinium (Gd)-enhanced contrast dye gadopentetate dimeglumine ((GdDTPA), 10% in phosphate-buffered saline (PBS)) was subcutaneously injected. Imaging was performed with a 35 mm mouse brain volume coil. Images were then obtained after injection, and dye was visualized with T1-weighted RARE sequencing with the following parameters: FOV: 3.0 cm × 3.0 cm, matrix: 256 × 256, TR: 1650 ms, TE: 10.6 ms, Inversion Time: 650 ms, averages: 1, Rare Factor: 2, slice thickness: 1 mm, inter-slice distance: 1 mm. Since Gd-DPTA clears quickly from the body and is non-toxic, we used the same animals (*n* = 3) to longitudinally analyze BBB permeability, allowing for minimization of inter-subject variability. On each MRI day, we performed a prescan prior to GdDTPA injection to ensure that no residual signal remained from the prior GdDTPA injections. Multiple scans were collected for each mouse up to 90 min post-GDDTPA injection, and the scans shown in this manuscript were collected 1 h after the GdDTPA injection. Sequential images through the lesion site were analyzed using ImageJ software (National Institutes of Health, Bethesda, MD). Total Gd signal through the lesion was quantified for each mouse from the ipsilateral cortex and expressed as percent of the corresponding Gd signal from the contralateral cortex.

### BBB integrity assay- Evans blue

Evans blue (EB) extravasation assessed BBB permeability following CCI. Two hours before euthanasia, animals were administered 200 μL of EB dye (2% *w*/*v*, i.p., Sigma-Aldrich, St Louis, MO) in saline. After euthanasia, animals were immediately transcardially perfused with ice-cold PBS, brains extracted and micro-dissected to obtain the cortex and thalamus/striatum in both the ipsilateral and contralateral hemispheres. Samples were weighed and placed in ice-cold trichloroacetic acid (50% w/v in distilled water), before homogenization by sonication. Homogenates were incubated at 4 °C for 30 min, followed by centrifugation at 16,000×g for 30 min at 4 °C. The supernatant of each sample was removed and absorbance measured at an excitation of 540 nm and an emission of 680 nm. Absorbance measurements were adjusted for tissue weight and subsequent levels of EB in each sample was calculated relative to a standard curve of known EB concentrations.

### Protein extraction

Subcellular fractions containing soluble and membrane bound proteins were separated by sequential centrifugation. Mice were perfused with ice-cold PBS; brain removed and a 5 mm-diameter region of tissue surrounding the cortex lesion, or tissue corresponding to the area of lesion in sham mice, was excised by punch extraction. Ipsilateral cortex punches were homogenized in PBS with protease and phosphatase inhibitors using a glass dounce (88668, Pierce, Waltham, MA), then centrifuged at 135,000 x g for 45 min at 4 °C. Following centrifugation, the supernatant containing PBS soluble proteins was removed and stored at − 80 °C. The remaining pellet was resuspended in ice-cold RIPA buffer containing protease and phosphatase inhibitors (20–188, Millipore, Billerica, MA), sonicated and centrifuged at 14,000 x g for 15 min at 4 °C. The subsequent RIPA supernatant containing membrane bound proteins was removed and stored at − 80 °C.

### Western blot analysis

Protein concentrations were measured using the BCA protein assay kit (23227, Pierce), with 20 μg of protein added to a 1:1 *v*/v of reducing buffer (2× Laemmli sample buffer (1610737, BioRad, Hercules, CA), 5% v/v β-mercaptoethanol (1610710, BioRad)) and heated at 95 °C for 5 min. Proteins were separated using SDS-PAGE gel electrophoresis and transferred to nitrocellulose membranes. Following transfer, membranes were blocked in 5% *w*/*v* skim milk powder in PBS-T (PBS, 0.05% v/v Tween-20, pH 7.6) for 1 h. Blots were then incubated with primary antibodies in 2% w/v skim milk/TBS-T, overnight at 4 °C. Primary antibodies used were PDGFRβ (1:1000, #3169, Cell Signaling, Danvers, MA), apoE (1:1000, ab1907, Abcam, Cambridge, MA), MMP-9 (1:800, 38898, Abcam) and β-Actin (1:5000, A5441, Sigma-Aldrich). Following primary antibody incubation, membranes were washed for 3 × 10 min in PBS-T at RT, before incubation with HRP-conjugated goat anti-rabbit, goat anti-mouse or rabbit anti-goat (Jackson ImmunoResearch, Westgrove, PA, 1:1000 in 2% w/v skim milk powder in PBS-T) secondary antibodies, for 90 min at RT. Membranes then washed for 3 × 10 min in PBS-T before detection using SuperSignal West Pico Chemiluminescent Substrate detection kit (34580, ThermoScientific, Waltham, MA) and visualization with Amersham 600 imaging machine (GE healthcare, Chicago, IL). Raw pixel intensities of bands from Western blots were quantified by densitometry using Image J software (Version 1.47, NIH, Bethesda, MD).

### Immunohistological analysis

Free-floating parallel brain sections (20 μm thickness) from wildtype, APOE3, and APOE4 mice were cut using a microtome (Microm HM 430, Thermo Fisher Scientific, Tusin, CA), and washed three times with PBS, before staining and incubated with blocking buffer (5% normal goat serum (NGS) in PBS) and 0.3% Triton X-100 (PBST) for 2 h at room temperature. Brain sections were incubated overnight at 4 °C with the following primary antibodies: rabbit polyclonal PDGFRβ antibody for pericytes (1:200, #3169, Cell Signaling), dylight 488 labeled *Lycopersicon Esculentum* (tomato lectin) (1:1000, DL-1174, Vector Laboratories, Burlingame, CA) for blood vessels, rabbit anti-CD31 (1:500, ab28364, Abcam) for microvessels, mouse monoclonal anti-Claudin-5 antibody (1:500, 35–2500, Invitrogen, Carlsbad, CA) and rabbit anti-Zonula Occludens 1 (1:500, 21,773–1-AP, Proteintech, Rosemont, IL) for tight junctions in the epithelial cells. After three 5 min washes in PBS, sections were incubated for 2 h with a corresponding anti-rabbit Alexa Fluor 568-conjugated or anti-mouse Alexa Fluor 594-conjugated IgG secondary antibodies (1:1000, S11227, Invitrogen) for 2 h at room temperature. As negative controls, sections were incubated without primary antibody. Sections were rinsed with PBS and distilled water and coverslipped with Fluoro-Gel with Tris Buffer mounting medium (Electron Microscopy Sciences, Hatfield, PA). Quantitative image analysis of the PDGFRβ Claudin-5, Zonula Occludens-1 and CD31 immunoreactive cortical regions were performed on five fields (× 20, 151.894 mm^2^), and three cortical sections per animal through the level of impact site using the same densitometric analysis method as previously described [23]. Image J software was used for analysis as previously described [23].

### In-situ Zymography

Following euthanasia and transcardial perfusion with ice-cold PBS, brains were harvested and flash frozen on dry ice. Brains were cryosectioned into 20 μm sections in series that encompass the ipsilateral lesion, as well as the contralateral hemisphere. Fluorescein conjugated, dye-quenched gelatin (DQ-gelatin, 1 mg/ml, D12054, Molecular Probes, Invitrogen) was prepared in reaction buffer (50 mM Tris HCl, Tris Base NH_2_C(CH_2_OH)_3_, 150 mM NaCl, 5 mM CaCl_2_, 0.2 mM NaN_3_, pH 7.6) and stored at 4 °C. Sections were incubated in DQ-gelatin reaction buffer in the dark for 1 h at 37 °C. Following incubation, reaction buffer was removed and coverslips placed over the tissue using vectashield. Fluorescence visualized using an Olympus BX51 microscope (Olympus American, Center Valley, PA) equipped with Olympus DP72 camera, and cell Sens Standard software.

### In situ hybridization combined with immunohistochemistry

Coronal brain sections (20 μm thickness) were washed three times in PBS before mounting on gelatin-coated glass slides (Superfrost Plus, Thermo Fisher Scientific) and stored at − 80 °C until use. Tissue was allowed to dry at room temperature (RT) and then stored at -20 °C until use. Fluorescent in situ hybridization (FISH) was performed using RNAscope® Technology 2.0 (Advanced Cell Diagnostics (ACD), Hayward, CA), as previously described [23, 25]. In short, mounted tissue sections were serially dehydrated in 50%, 70%, 95%, 100% and 100% ethanol for 5 min each. In between all pretreatment steps, tissue sections were briefly washed with ultra-pure water. The pretreat solution 1 (hydrogen peroxide reagent) was applied for 10 min at RT, and then the tissue sections were boiled in pretreat solution 2 (target retrieval reagent) for 15 min. Mounted slices were treated with pretreat solution 3 (protease reagent) for 30 min at 40 °C on the HybEz™ hybridization system (ACD). Mouse Apoe probe (Cat. No. 313278, ACD) targets the region 83–1245 (Accession number: NM_009696.3) of the Apoe sequence with 19 pairs of ZZ-target probes. In addition, the negative (Cat. No. 310043, ACD) and positive (Cat. No. 313911, ACD) control probes were applied and let hybridized for 2 h at 40 °C. The amplification steps were performed according to manufacturer’s directions. In between every amplification step, sections were washed with 1× wash buffer. Detection was performed using a mixture ratio of the Red-A to the Red-B solution of 1:60. Sections were incubated for 10 min at RT and rinsed with ultra-pure water. Following in situ hybridization, the sections were processed for immunohistochemistry. Briefly, following the blocking step with 5% NGS in PBS for 1 h at RT, post-hybridized slides were incubated with an antibody against anti-mouse GFAP (glial fibrillary acidic protein, 1:1000, EMD Millipore, Temecula, CA) or anti-rabbit PDGFRβ in the presence of 2% NGS in PBS overnight at 4 °C. After three washes with PBS, the slides were incubated with corresponding anti-mouse or anti-rabbit Alexa Fluor® 488 secondary antibodies (1:500; Molecular probes®, ThermoFisher Scientific, Grand Island, NY) for 2 h at RT. Brain sections were rinsed with PBS three times and incubated for 5 min in PBS with DAPI solution (1: 50,000, Sigma-Aldrich) for counterstained nuclei. Fluorescent images were acquired on an Olympus XB51 microscope equipped with Olympus DP72 camera, and cell Sens Standard software.

### Microvessel isolation

Microvessels were isolated as previously described [26]. Briefly, sham and CCI mice were euthanized and ipsilateral and contralateral brain tissue punch excised and rinsed in sucrose buffer (S.B, 0.32 mol/L sucrose, 3 mmol/L HEPES, pH 7.4). Samples were homogenized in a glass dounce homogenizer containing 2 ml S.B., then centrifuged for 10 min × 1000 *g* at 4 °C. Following centrifugation, the supernatant (containing the visible white upper myelin layer) was discarded and remaining pellet resuspended in 2 ml S.B., re-homogenized using glass dounce and centrifuged again for 10 min × 1000 *g* at 4 °C. Supernatant was discarded and the sediment pellet was resuspended in a further 2 ml S.B. and centrifuged (30s × 100 *g,* 4 °C). Following centrifugation, supernatant was removed and kept, and the resulting pellet underwent a second resuspension (in 2 ml S.B) and centrifuged (30s × 100 *g,* 4 °C). The supernatant was removed and pooled together, before a final centrifugation for 2 min × 200 *g* at 4 °C. The resulting pellet was resuspended (25 μl, 0.1% BSA in PBS) and microvessels confirmed by Toluidin Blue staining and brightfield microscopy, before being prepared for RNA extraction.

### RNA isolation and cDNA synthesis

RNA was extracted from microvessels using TRIzol® reagent (15596026, Invitrogen) as per manufacturer’s guidelines. Concentration and purity of the RNA was assessed using the NanoDrop 1000 spectrophotometer (ThermoScientific). One microgram of RNA was reverse transcribed into cDNA using a High-Capacity RNA-to-cDNA Reverse Transcription Kit (4368814, Applied Biosystems, Foster City, CA) in accordance with manufacturer’s guidelines. The resulting cDNA was diluted 1:3 in diethylpyrocarbonate (DEPC) treated H_2_O for use in RT-QPCR. Samples were stored at − 80 °C until use.

### Real time quantitative polymerase chain reaction (RT-QPCR)

RT-QPCR was conducted as described previously [27, 28]. Briefly, RT-QPCR was performed in triplicate in standard 384-well plates using the Prism 7900HT fast sequence detection system (Applied Biosystems). Taqman probes were used to analyze *GAPDH* (Mm99999915_m1), *apoe* (Mm01307193_g1), *APOE* (Hs00171168_m1), *Mmp-9* (Mm01317678_m1), *Claudin-5* (Mm00727012_s1), *Occludin* (Mm00500912_m1), *Zonula Occludens-1* (Mm00493699_m1) and *Pdgfrβ* (Mm00435546_m1) under the following cycle conditions, 50 °C for 2 min, 95 °C for 20 s, (95 °C for 1 s, 60 °C for 20 s) × 40 repeats. The SDS 2.4 software (Applied Biosystems) was used to generate threshold cycle (Ct) values, and fold change in mRNA expression calculated using the ΔΔCt method (2-ΔΔCt) as previously described [29].

### Statistical analysis

All data is presented as mean ± SEM. Data was analyzed using a One-way ANOVA with Dunnett’s post hoc test or a Two-way ANOVA with Bonferroni multiple comparison post-hoc tests where appropriate. All statistical analysis was carried out using Graph Pad Prism™ Software (version 7.0), with *p < 0.05 considered statistically significant for all experiments.

## Results

### Characterization of BBB permeability and stabilization following TBI

**Fig. 1:**
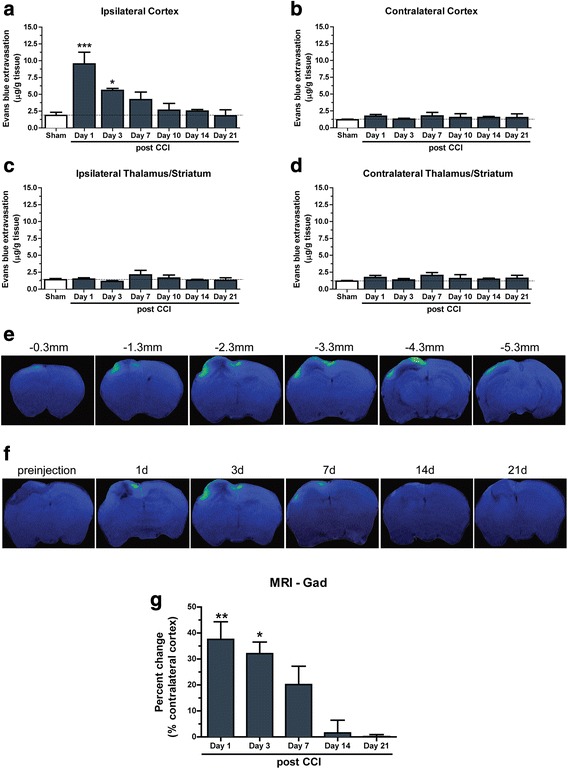
Characterization of BBB permeability following TBI. (**a**) Increased EB extravasation in the ipsilateral cortex of wildtype mice at 1 (***p < 0.001, *p < 0.05; *n* = 4) and 3 days post-injury compared to sham. No change is observed in the (**b**) contralateral cortex (*n* = 4), (**c**) ipsilateral Thalamus/Striatum (*n* = 4) or (**d**) contralateral Thalamus/Striatum (*n* = 4) compared to sham. (**e**) T1-weighted MRI images representing the range of pericontusional leakage across bregma brain coordinates of a single wildtype mouse, at 3 days post-TBI. (**f**) T1-weighted MRI images displaying wildtype mice pre-injection and at 1, 3, 7, 14 and 21 days post-TBI. (**g**) Quantification of the T1-weighted MRI time course of BBB leakage post TBI ((**p < 0.01, *p < 0.05 vs Day 14 and Day 21; *n* = 3 per group). The intensity of signal increases from blue<green. Data expressed as mean ± S.E.M. One way ANOVA with Dunnett post hoc test

It has been well established that BBB dysfunction occurs following TBI, contributing to secondary injury processes and the overall degree of injury severity [30]. To characterize the time course of BBB permeability following primary impact, wildtype mice were subject to TBI before BBB permeability assessed by EB analysis. Levels of EB extravasation were increased by 411% in the ipsilateral cortex at day 1 (*P* < 0.001) and remained elevated by 201% at day 3 post-injury (*P* < 0.05) compared to sham animals (Fig. 1a). Full closure of the BBB to EB permeability was observed approximately 7–10 days post-injury. No change in BBB permeability was observed in the contralateral cortex (Fig. 1b) or the ipsilateral or contralateral thalamus/striatum at any timepoint post-TBI (Fig. 1c-d).

We also assessed BBB permeability using GdDTPA enhanced MRI. Consistent with EB observations, T1-weighted MRI with Gadolinium-enhanced contrast displayed increased leakage in the pericontusional area of the ipsilateral cortex that extended approximately 5 mm from the rostral to the caudal aspect of the lesion (Fig. 1e). We assessed GdDTPA leakage into the mouse brain at multiple timepoints up to 21 days post-TBI and observed GdDTPA signal at 1, 3, and 7 days post-injury compared to pre-injection scans, but not at 14 or 21 days post-injury (Fig. 1f). We quantified this signal and confirmed that the BBB was fully closed to GdDTPA leakage at 14d post-TBI (Fig. 1g).

### Levels of soluble apoE are inversely associated with MMP-9 activation

**Fig. 2:**
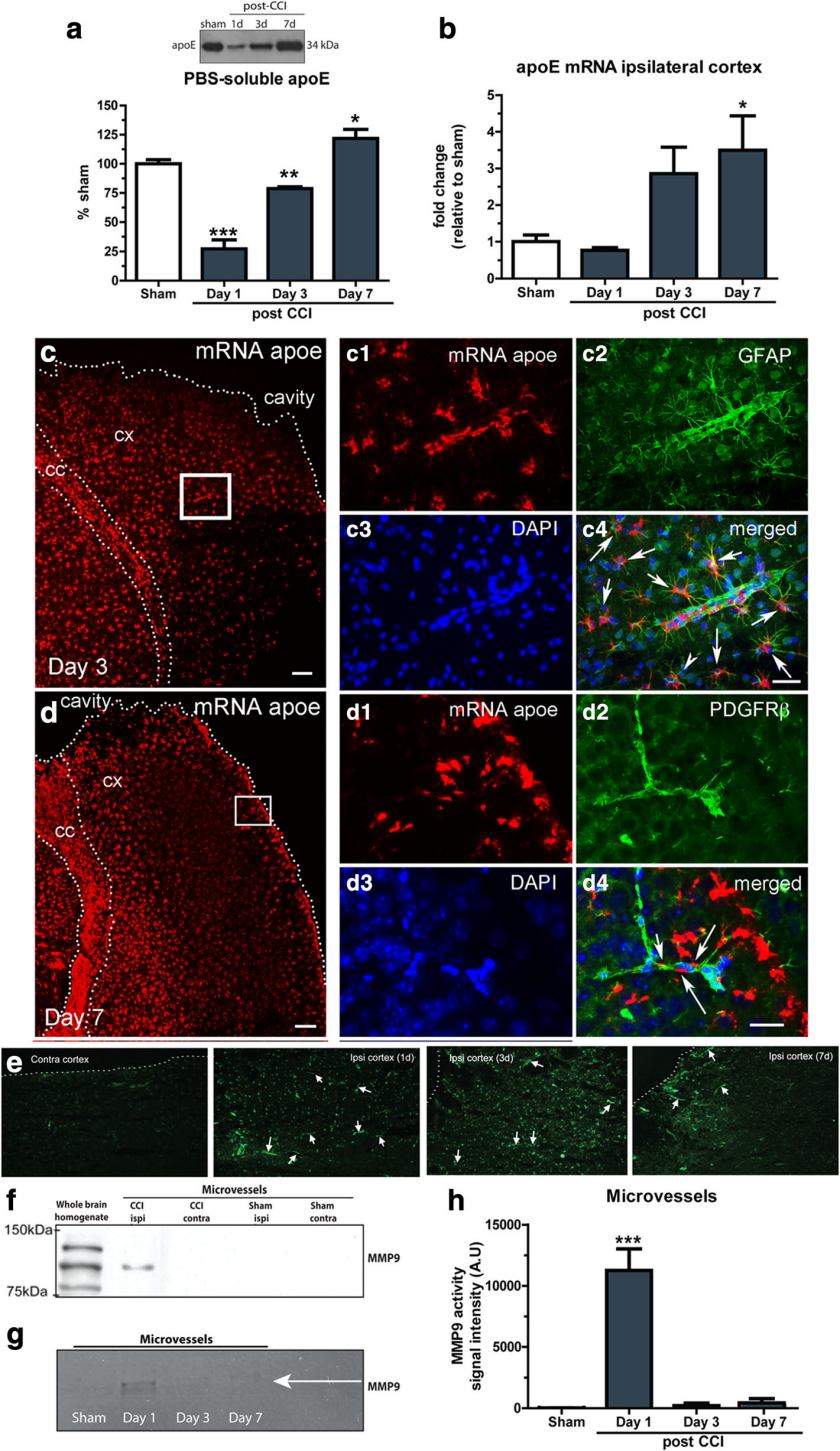
TBI-induced apoE and MMP-9 responses in the ipsilateral cortex of wildtype mice. Wildtype mice were subject to TBI before whole ipsilateral cortex punches collected at 1, 3, and 7 days post-injury for protein and RNA analysis. (**a**) Western blot and densitometry analysis of PBS-soluble apoE in the ipsilateral cortex of wildtype mice following TBI (*n* = 3, *p < 0.05, **p < 0.01, ***p < 0.001). (**b**) *apoe* mRNA expression post-injury (*n* = 4, *p < 0.05). (**c-d**) Fluorescent in situ hybridization analysis of apoe expression at 3 and 7 days post-TBI. (**c1–4**) *apoe* mRNA and GFAP immunofluorescence surrounding a cortical microvessel. (**d1–4**) *apoe* mRNA with PDGFRβ immunofluorescence. Scale bars C-D images 50 μm, inset C1-D4 images 20 μm. (**e**) In-situ zymography evidencing MMP-9 activity after TBI. (**f**) Western blot of MMP-9 in whole brain ipsilateral cortex and ipsilateral microvessels from wildtype mice 24 h post-injury (representative of n = 3). (**g**) In-gel zymography of MMP-9 activity in ipsilateral microvessels isolated from wildtype mice following TBI. (**h**) Densitometry analysis of in-gel MMP-9 zymography (*n* = 3, ***p < 0.001). Data expressed as mean ± S.E.M. One way ANOVA with Dunnett post hoc test

The apoE protein has been demonstrated to modulate cerebrovascular integrity at the NVU via an apoE-LRP1 mediated suppression of MMP-9 [9]. To characterize the apoE and MMP-9 response following TBI stimulus, wildtype mice were subject to TBI before a micropunch of the ipsilateral cortex was extracted and analyzed at 1, 3 and 7 days post-injury. TBI induced a biphasic response in PBS-soluble apoE protein (Fig. 2a), with protein levels significantly reduced by 73% at day 1 (*P* < 0.001) and by 21% at day 3 (*P* < 0.01) post-injury compared to sham (Fig. 2a). At 7 days post-injury, apoE protein levels were significantly increased by 22% compared to sham levels (*P* < 0.05; Fig. 2a). *Apoe* mRNA expression in cortical punch was elevated by 3.5-fold in the ipsilateral cortex at 7 days post-injury (P < 0.05) (Fig. 2b). To explore the source of *apoe* mRNA after TBI, we used *apoe* mRNA FISH alongside markers of astrocytes and pericytes. We found widespread expression of apoe in our mice, with the majority of expression found in GFAP-positive astrocytes (Fig. 2c). Fig. 2C1–4 show *apoe* mRNA and GFAP immunofluorescence surrounding a cortical microvessel. The astrocytic projections and endfeet surrounding the microvessel are clear, however it is also apparent that cells associated with the microvessel are also producing *apoe* mRNA. To investigate this further, we repeated our *apoe* mRNA FISH with PDGFRβ immunofluorescence. We found colocalization of the PDGFRβ positive cells with *apoe* mRNA (Fig. 2d, D1–4).

To determine if microvessels were locally producing MMP, we used in-situ zymography. We report that MMP activity was greatly increased in the ipsilateral cortex at 1 days post-TBI, and that microvessel-related MMP activity was clearly visible in the ipsilateral cortex using this technique (Fig. 2e, arrows indicate microvessel-associated activity). At 3 days post-injury MMP activity was still visible, but there were noticeably less microvessel-associated activity compared to 1 day post-TBI. At 7 days post-injury there was still ongoing microvessel associated activity, but this was spatially limited to the area directly adjacent to the lesion (Fig. 2e). We next isolated microvessels from micropunches of the ipsilateral and contralateral cortex of TBI and sham mice and analyzed these vessels for the presence of MMP-9 protein. MMP-9 protein was detectable in the ipsilateral cortex microvessels from TBI mice, but not from the contralateral microvessels, or from sham microvessels (Fig. 2f). We further performed gel zymography on isolated microvessels from the ipsilateral cortex at 1, 3, and 7 days post-TBI, and found that while TBI substantially increased microvessel MMP-9 activity at 1 day post-TBI, but that this activity was below detection threshold at 3 and 7 days post-injury (Fig. 2g-h).

### Characterization of the NVU response in wildtype mice following TBI

**Fig. 3:**
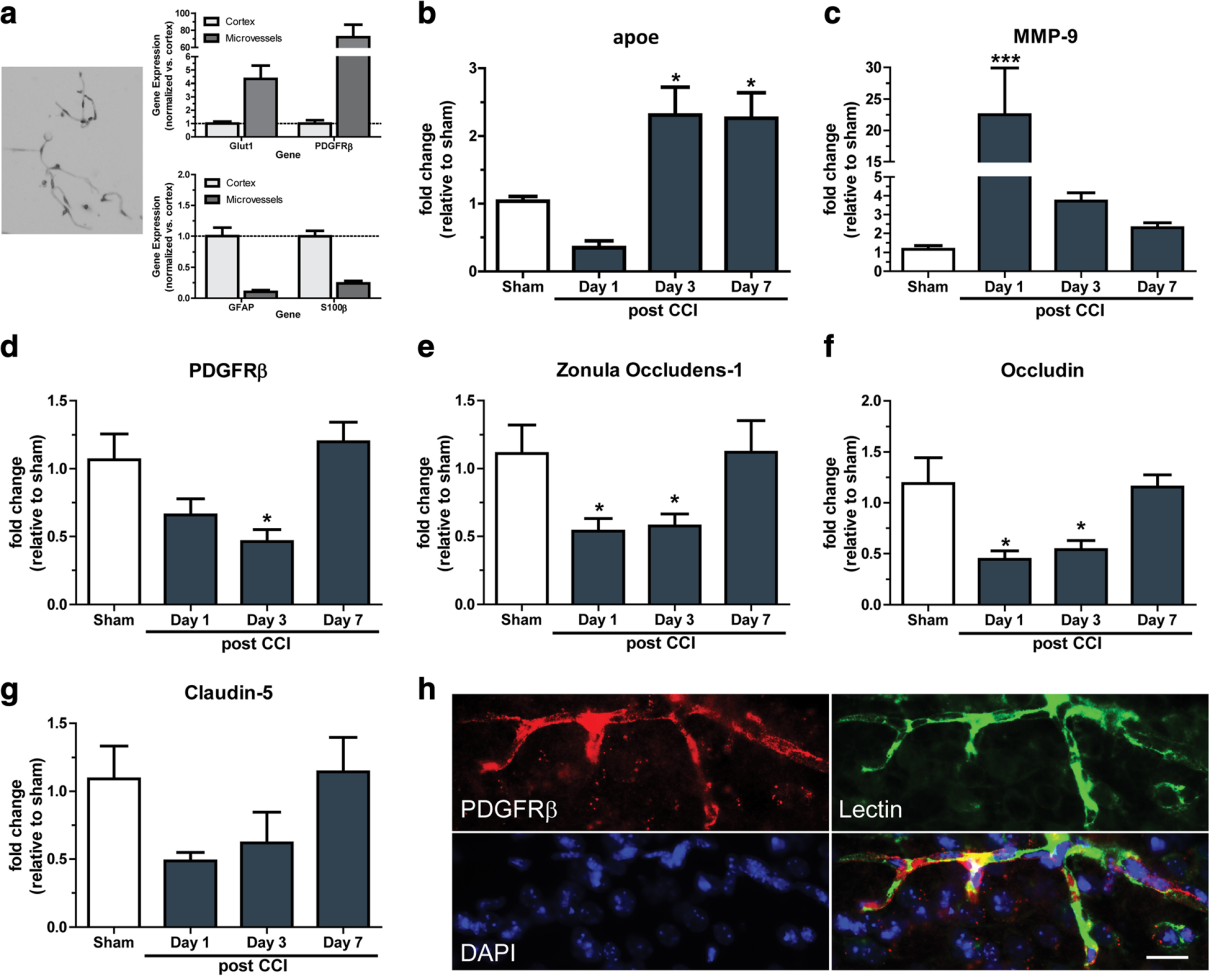
Characterization of the NVU response in wildtype mice following TBI. (**a**) Representative image of toluidine blue stained microvessels isolated from the ipsilateral cortex. RT-QPCR analysis shows the purity of microvessel preparations, consisting of enriched endothelial (Glut1) and pericyte (PDGFRβ) markers with minimal astrocytic expression (GFAP/S100β) in comparison to whole cortex punch fractions. Wildtype mice were subject to TBI before microvessels isolated at 1, 3 and 7 days post-injury and subject to RNA analysis. RT-QPCR analysis of the mRNA expression for (**b**) *apoe* (*n* = 3, *p < 0.05), (**c**) *Mmp-9* (*n* = 5–6, ***p < 0.001), (**d**) *Pdgfrβ* (*n* = 6, *p < 0.05), (**e**) *Zonula Occludens-1* (*n* = 5–6, *p < 0.05), (**f**) *Occludin* (*n* = 5–6, *p < 0.05) and (**g**) *Claudin-5* (Sham, day 1, day 3 *n* = 5–6, day 7 *n* = 3). (**h**) Immunohistochemistry analysis assessing the co-localization of the respective microvessel and pericyte markers, Lectin and PDGFRβ. Scale bar: 20 μm. Data expressed as mean ± S.E.M. One way ANOVA with Dunnett post hoc test

The NVU is a crucial structure that regulates BBB vascular integrity, stability and maintenance. To further elucidate the specific TBI mediated BBB response at the NVU, wildtype mice were subject to TBI before ipsilateral cortex microvessels were isolated and analyzed by RT-QPCR. Baseline characterization of microvessel preparations from sham mice revealed elevated *Glut1* and *PDGFRβ* mRNA expression, indicative of enriched endothelial and pericyte populations compared to whole cortex tissue (Fig. 3a). Furthermore, these microvessels contain minimal astrocytic presence, with reduced levels *GFAP* and *S100β* observed compared to whole cortex fractions (Fig. 3a). Following TBI, *apoe* mRNA expression was significantly increased at 3 and 7 days post-injury compared to sham (*P* < 0.05; Fig. 3b). *Mmp-9* expression was significantly increased 1 day post-injury (*P* < 0.001; Fig. 3c), but returned towards baseline by 3 days post-injury. There was a time-dependent decrease in *Pdgfrβ* mRNA, a pericyte marker, until 3 days post-TBI (P < 0.05), before recovering at day 7 post-injury (Fig. 3d). At the tight-junction protein level, we observed significant decreases in mRNA for *Zonula Occludens-1* and *Occludin* at 1 and 3 days post-injury (*P* < 0.05; Fig. 3e, f), with a similar trend in the mRNA for *Claudin-5* (Fig. 3g). All tight-junction protein mRNA returned to baseline expression by 7 days post-injury. We confirmed that PDGFRβ is found on vessels by examining protein co-localization with vessel specific lectin and examining vessel size and morphology. PDGFRβ colocalizes with lectin, and was found primarily on vessels with a diameter of 5–20μM (Fig. 3h).

### Treatment with the Cyclophilin A (CypA) antagonist Cyclosporine A (CsA) improves BBB repair after TBI

**Fig. 4:**
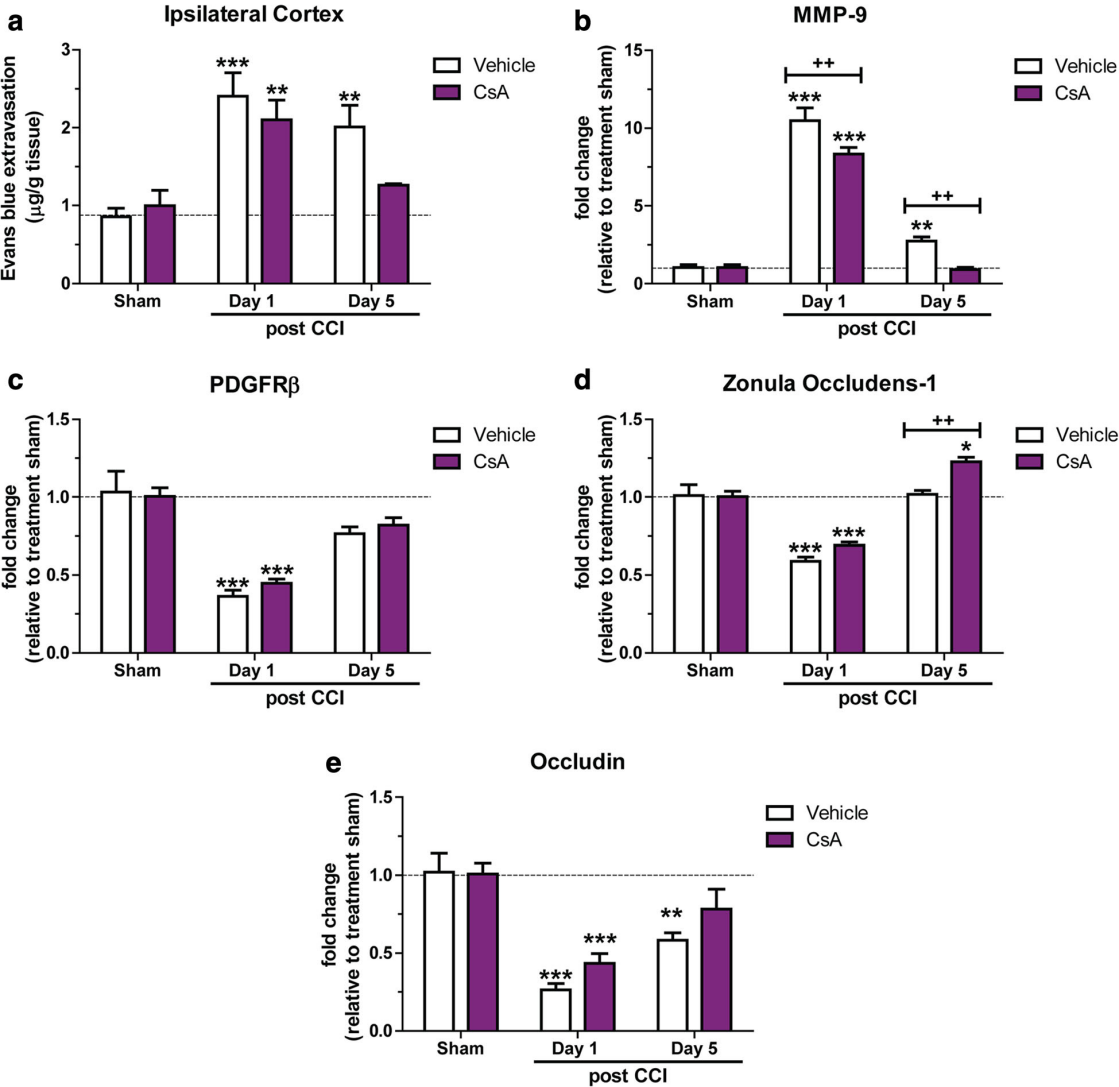
Cyclosporine A (CsA) treatment improves BBB stabilization and closure in wildtype mice. Wildtype mice were subject to TBI before administration of 20 mg/kg CsA or vehicle at 15 min post-injury. (**a**) Evans blue analysis of BBB permeability in the ipsilateral cortex analyzed at 1 and 5 days post-injury (*n* = 5 per group and timepoint, ***p < 0.001, **p < 0.05). Isolated microvessels analyzed by RT-QPCR for (**b**) *Mmp-9* (*n* = 4, ***p < 0.001, **p < 0.01 vs treatment sham, ^++^*p* < 0.01 vs relevant timepoint), (**c**) *Pdgfrβ* (*n* = 4, ***p < 0.001) (**d**) *Zonula Occludens-1* (*n* = 4, ***p < 0.001, *p < 0.05 vs treatment sham, ^++^*p* < 0.01 vs relevant timepoint) and (**e**) *Occludin* (*n* = 4, ***p < 0.001, *p < 0.05) mRNA expression. All data expressed as mean ± S.E.M. Two-way ANOVA with Bonferroni post hoc test

To investigate the role of CypA–MMP-9 in BBB stabilization and repair, wildtype mice were subject to TBI, followed by administration of either CsA or vehicle. We assessed acute BBB permeability at 1 day to establish if CypA was involved in the acute response to trauma, and at 5 days post-injury to assess if inhibition of CypA could induce faster BBB stabilization than the 7–10 day spontaneous closure window we observed in untreated mice. At 1 day following TBI, EB extravasation from the ipsilateral cortex was significantly elevated in both vehicle and CsA treated mice (*P* < 0.001). At 5 days post-injury, BBB permeability remained significantly elevated by 135% in vehicle treated mice (*P* < 0.01 vs vehicle sham), but had returned to sham levels in CsA treated animals (P < 0.05 vs. CsA sham mice, Fig. 4a). In a separate cohort of mice, the effect of CsA on NVU responses was investigated by isolating microvessels from the ipsilateral cortex of CsA and vehicle treated wildtype mice after TBI. RT-QPCR analysis showed *Mmp-9* expression was increased in the vehicle group at 1 and 5 days post-injury, a response that is significantly attenuated by CsA administration (P < 0.01; Fig. 4b). Pericyte expression was unchanged, with *Pdgfrβ* mRNA reduced in both groups at 1 day post-TBI (*P* < 0.001, Fig. 4c), before rebounding by day 5 post-injury. At the tight junction level, we observed significant decreases in mRNA for *Zonula Occludens-1* and *Occludin* in both CsA and vehicle groups at 1 day post-TBI (P < 0.001; Fig. 4d, e). However, at 5 days post injury, CsA treatment resulted in a 20% increase in *Zonula Occludens-1* (P < 0.001; Fig. 4d), whilst *Occludin* levels were unchanged to that observed in sham animals.

### APOE4 mice exhibit impaired closure of the BBB and delayed pericyte recovery after TBI

**Fig. 5:**
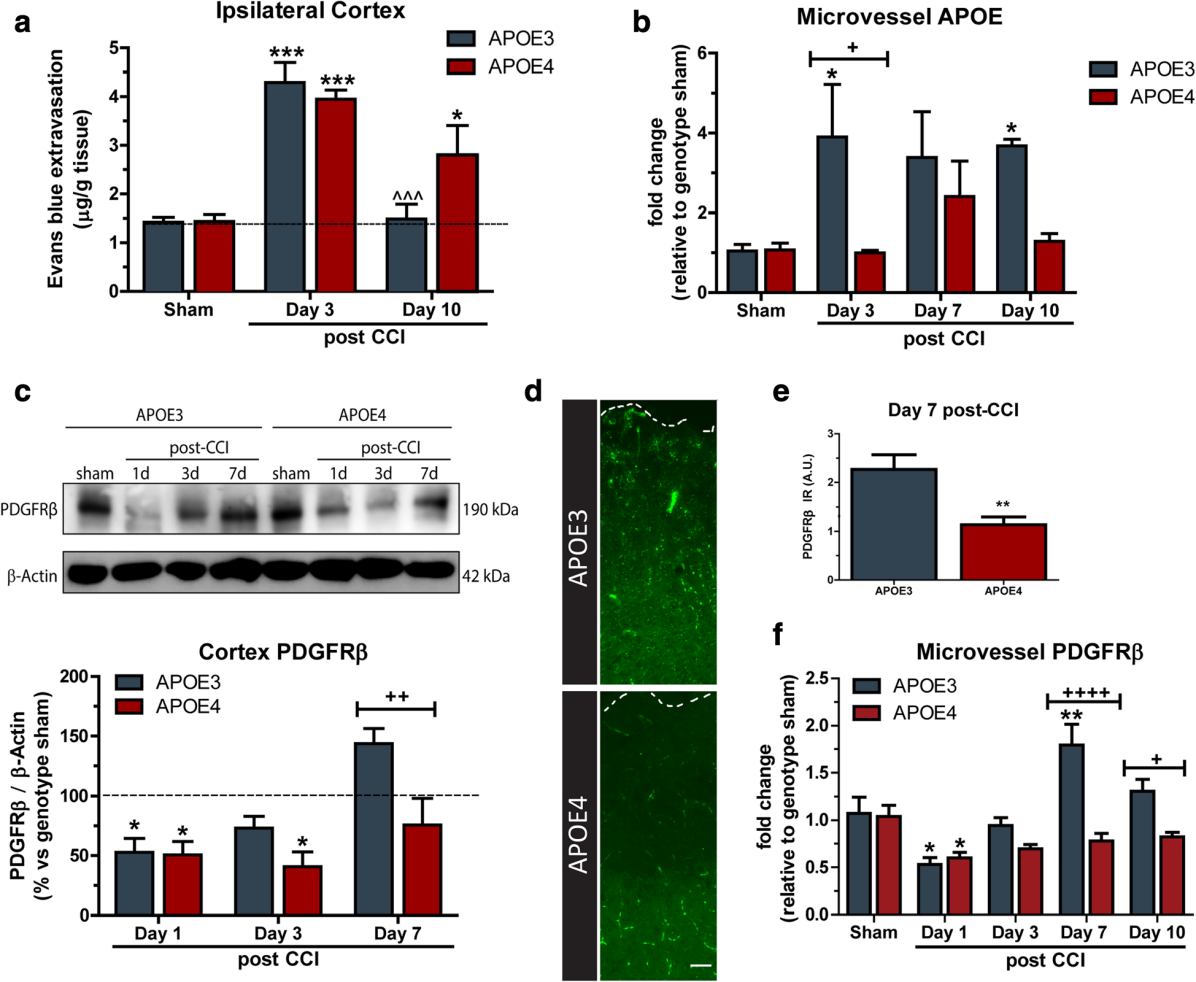
APOE4 mice exhibit impaired closure of the BBB and delayed pericyte recovery after TBI. (**a**) APOE3 and APOE4 mice were subject to TBI, before BBB permeability in the ipsilateral cortex analyzed at 3 and 10 days post-injury using Evans Blue extravasation (*n* = 5, ***p < 0.001, *p < 0.05 vs genotype sham, ^^^*p* < 0.001 vs APOE3 day 3). (**b**) APOE mRNA expression in isolated microvessels (*n* = 4–6, *p < 0.05 vs genotype sham; ^+^*p* < 0.05 vs relevant timepoint). (**c**) Western blot and densitometry analysis of PDGFRβ expression in the ipsilateral cortex of APOE3 and APOE4 mice following TBI (*n* = 4, *p < 0.05 vs genotype sham; ^++^*p* < 0.01 vs relevant timepoint). (**d-e**) Immunohistochemistry displaying reduced PDGFRβ expression in the ipsilateral cortex of APOE4 compared with APOE3 mice at 7-days post-TBI. Scale bar: 200 μm. (*n* = 4 per genotype, **p < 0.01). (**f**) *Pdgfrβ* mRNA expression in microvessels isolated from ipsilateral cortex of APOE3 and APOE4 mice after TBI (*n* = 4–6, **p < 0.01, *p < 0.05 vs genotype sham; ^++++^*p* < 0.0001, ^+^*p* < 0.05 vs relevant timepoint). Data expressed as mean ± S.E.M. Two-way ANOVA with Bonferroni post-hoc test

The human apoE4 protein is known to be functionally deficient compared to the apoE3 protein, and we used humanized APOE mice as a clinically relevant model to study the role of apoE on BBB injury and repair after TBI. APOE3 and APOE4 mice were subject to TBI and their BBB permeability assessed using the EB method. 3 days following TBI, EB extravasation from the ipsilateral cortex was increased by 202% in APOE3 mice (*P* < 0.0001 vs APOE3 sham) and by 174% in APOE4 mice (P < 0.001 vs APOE4 sham) (Fig. 5a). Similar to wildtype mice, BBB permeability in APOE3 TBI mice had returned to within 5% of APOE3 sham levels by 10 days post-TBI, and was significantly reduced compared to APOE3 mice at 3 days post-TBI (*P* < 0.001). In contrast, BBB closure to EB was not achieved in APOE4 mice at 10 days post-TBI. At 10 days post-injury, BBB permeability in APOE4 mice remained significantly elevated by 94% compared to APOE4 sham mice (*P* < 0.05), and was not significantly decreased compared to APOE4 3 day mice (Fig. 5a). Previously we have demonstrated reduced levels of soluble apoE in the ipsilateral cortex of APOE4 mice compared to APOE3, up to 7 days post-TBI [8]. To investigate the NVU specific *apoe* response, ipsilateral microvessels were isolated and assessed after TBI. APOE3 microvessels displayed significantly elevated a*poe* mRNA expression at 3 and 10 days post-injury compared to APOE4 counterparts (P < 0.05; Fig. 5b). Given the role of brain pericytes in maintaining and regulating the integrity of the BBB [31, 32], we investigated the pericyte response to TBI in APOE3 and APOE4 mice. Western blot analysis of the ipsilateral cortex revealed that both APOE3 and APOE4 exhibit acute pericytes loss, with a 47% loss of PDGFRβ protein in APOE3 mice at 1 day post-TBI, and a 49% loss in APOE4 mice at the same timepoint (P < 0.05 vs genotype sham; Fig. 5c). PDGFRβ expression recovers by 3 days post-TBI in APOE3 mice, and is elevated by 44% at 7d post-TBI. In contrast, PDGFRβ remains significantly depressed at 3 days in APOE4 mice (*P* < 0.05 vs APOE4 sham), and remains 47% below APOE3 PDGFRβ levels at 7 days post-TBI (Fig. 5c, P < 0.01). In support of this finding, we found that immunohistochemistry for PDGFRβ was reduced in the pericontusional cortex of APOE4 mice compared to APOE3 mice at 7 days post injury (Fig. 5d-e). Furthermore, RT-QPCR analysis of the pericyte marker *Pdgfrβ* in isolated microvessels, revealed APOE4 animals exhibit decreased expression at 7 (P < 0.001, Fig. 5f) and 10 days post-injury (*P* < 0.05, Fig. 5f) in comparison to APOE3 mice.

### APOE4 mice display impaired BBB stabilization at the NVU following TBI

**Fig. 6:**
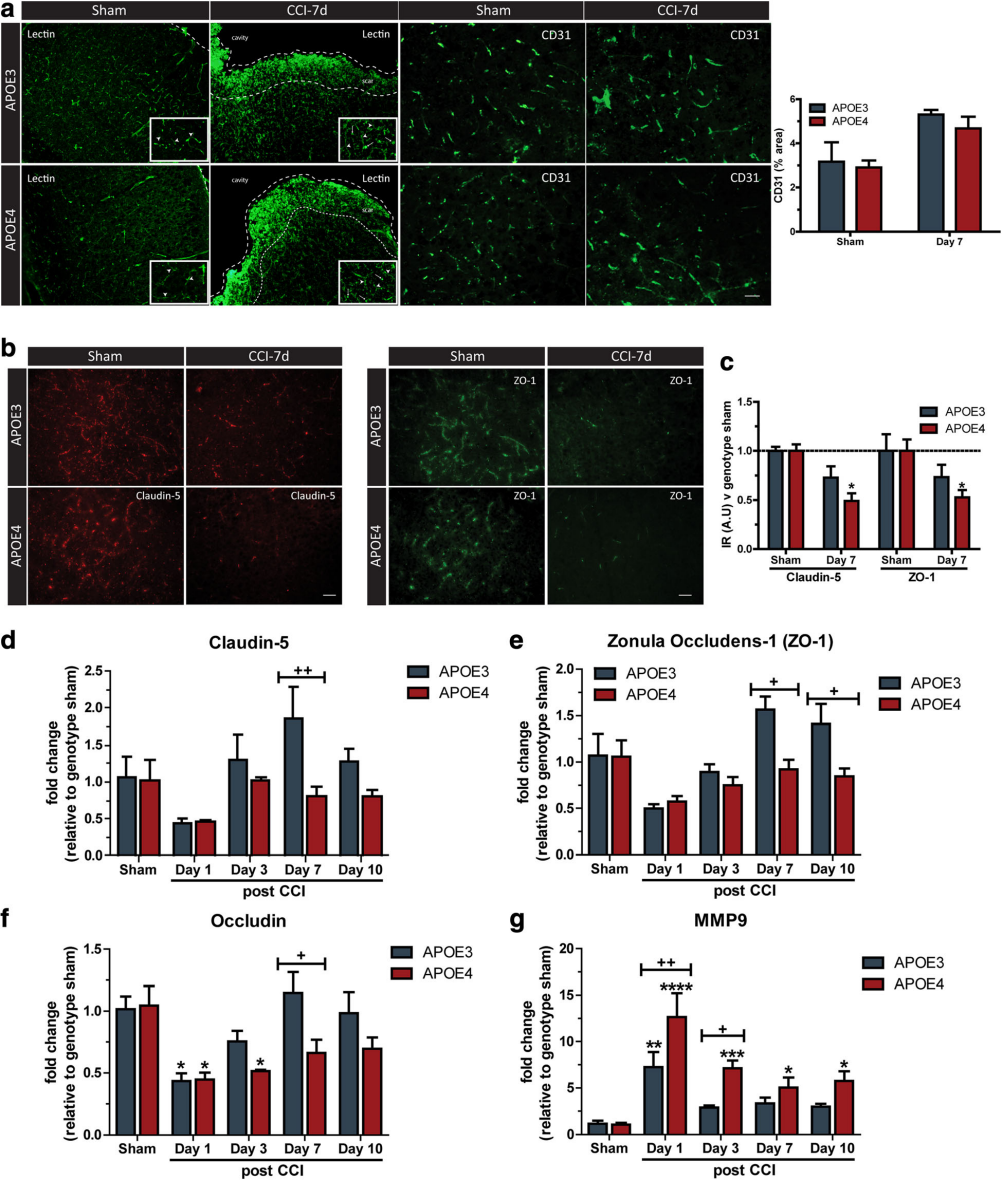
APOE4 mice display prolonged BBB dysfunction at the NVU following TBI. (**a**) Immunohistochemistry analysis of lectin (inset: arrowheads = vessel, arrows = glial) and CD31 staining in the ipsilateral pericontusional cortex following TBI (*n* = 3–4). (**b-c**) Quantitative immunohistochemistry showing the expression of Claudin-5 and Zonula Occludens-1 (ZO-1) in the ipsilateral pericontusional cortex of APOE3 and APOE4 sham and injured mice. (*n* = 4, *p < 0.05 vs genotype sham). APOE3 and APOE4 mice were subject to TBI, before ipsilateral microvessels were isolated at 1, 3, 7 and 10 days post-injury. mRNA analysis of (**d**) *Claudin-5* (*n* = 4–6, ^++^*p* < 0.01 vs relevant timepoint), (**e**) *Zonula Occludens-1* (*n* = 4–6, ^+^*p* < 0.05 vs relevant timepoint), (**f**) *Occludin* (*n* = 4–6, *p < 0.05 vs genotype sham; ^+^*p* < 0.05 vs relevant timepoint) and (**g**) *Mmp-9* (*n* = 4–6, ***p < 0.001, **p < 0.01, *p < 0.05, vs genotype sham. ^+^*p* < 0.05, ^++^*p* < 0.01 vs relevant timepoint). All data expressed as mean ± S.E.M. Two-way ANOVA with Bonferroni post hoc test

To investigate the genotype specific effects of APOE on BBB dynamics, APOE3 and APOE4 mice were subject to TBI before cerebrovascular integrity assessed. We used lectin and CD31 to determine if the number of blood vessels was impacted in the perilesion area 7 days post-TBI. In sham mice, we found no effect of genotype using either lectin or CD31. However we found that in TBI mice, lectin is no longer specific for blood vessels, with widespread cellular staining through the glial scar that had the morphological characteristics of glial cells (Fig. 6a). CD31 immunohistochemistry remained specific for blood vessels, and we found that neither APOE genotype nor TBI caused a significant change in the area of blood vessel coverage in the pericontusional area (Fig. 6a). At the tight junction level, immunohistochemistry analysis showed APOE4 mice display reduced levels of Claudin-5 and Zonula Occludens-1 at 7 days post-TBI compared to APOE3 mice (Fig. 6b, c). In support of our protein analysis, APOE4 microvessels isolated from a micropunch of the ipsilateral cortex display significantly reduced mRNA expression of *Claudin-5* (P < 0.05, Fig. 6d), *Zonula Occludens-1* (Day 3 and 10, P < 0.05, Fig. 6e) and *Occludin* (P < 0.05, Fig. 6f) after TBI compared to APOE3 counterparts. Furthermore, increased levels of *Mmp-9* is observed in APOE4 microvessels at day 1 (*P* < 0.01, Fig. 6g) and day 3 (P < 0.05, Fig. 6g) post-TBI compared to APOE3 microvessels. In addition, *Mmp-9* expression remained elevated in APOE4 microvessels at day 7 and day 10 (P < 0.05, Fig. 6g) post-injury, relative to genotype control.

## Discussion

**Fig. 7:**
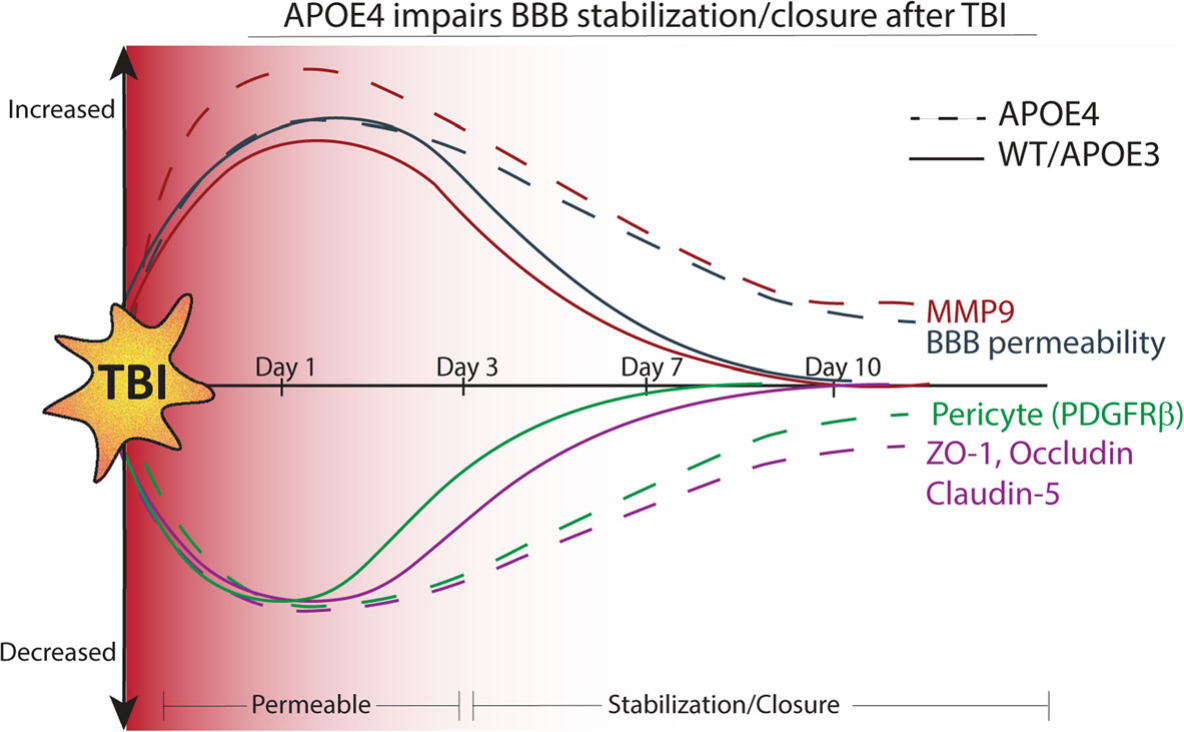
APOE isoforms influence the time course of BBB dysfunction following TBI**.** Diagram summarizing the time course of TBI-induced BBB disruption, stabilization and closure in Wildtype, APOE3 and APOE4 mice. Despite Wildtype, APOE3 and APOE4 mice all displaying similar acute BBB responses to TBI, such as elevated permeability and pericyte loss, APOE4 mice display a significantly extended period of BBB leakage following injury. This enhanced time-course of BBB permeability in APOE4 mice occurs in parallel with reduced pericyte repopulation back to the NVU after injury, as well as sustained MMP-9 responses that may cumulatively contribute to the prolonged levels of tight junction degradation

In this study, we characterized the extent of BBB permeability after TBI, and investigated the cellular and molecular processes underlying this pathology. Here we report TBI induces acute BBB dysfunction in C57Bl/6 mice, before stabilization at 7–10 days post-injury, a response that is inversely associated with soluble apoE levels. We used multiple techniques to demonstrate BBB dysfunction, including EB extravasation and GdDTPA MRI imaging. Cortical punch analysis of the ipsilateral cortex revealed an acute decrease in PBS-soluble apoE protein that rebounded to be significantly higher at 7d post-TBI, a timecourse that was inversely related to MMP-9 activity in microvessels isolated from the same area. RT-PCR analysis of isolated microvessels also revealed pericyte loss at 1 day post-TBI that corresponded with increased MMP-9 expression and a loss of tight junction protein expression. At 3-7d post-TBI we found that apoE expression is found locally at the NVU, and that this increase in expression corresponds with a return of pericytes to the NVU, decreased MMP-9 expression and activity, and stabilization and production of tight junction proteins (Fig. 7). FISH co-localization revealed that both astrocytes associated with the microvessel, and pericytes on the microvessel, express *apoe* mRNA. We report similar NVU responses to TBI in APOE3 mice, however we found that BBB stabilization was significantly delayed in APOE4 mice with a delayed pericyte repopulation, augmented and sustained MMP-9 expression at the NVU, and impaired stabilization of Zonula Occludens-1, Occludin and Claudin-5 expression at tight junctions after TBI, compared to APOE3 mice. Finally, we report prolonged BBB permeability in APOE4 but not APOE3 mice, showing that the E4 isoform prolongs BBB impairments following TBI.

It is clear that TBI can result in a loss cerebrovascular structure/integrity in both human and experimental TBI models. Post-mortem clinical evidence shows defined lesion alterations and reduced lumen vessels in cortical zones between 24 h-20 days post TBI [33]. Similar microvascular injury is observed almost universally in experimental TBI [34], including fluid percussion injury [35–38] and CCI models [39–42]. Ultrastructural and 3D microscopy analysis show pyknotic neurons, irreversible mechanically induced vascular damage, and morphological vessel alterations at the site of contusion acutely after injury [38, 43]. Indeed, the temporal progression of tissue loss at the cortical lesion is well characterized [41], however the impact of TBI on remaining pericontusional vascular structures is less well defined.

In remaining blood vessels, the importance of BBB repair in regaining vascular integrity is a well-established factor in reducing the impact of mediating secondary injury responses, disease severity and recovery timeframes [2]. Despite this, the extent and time course of TBI-associated BBB permeability remains poorly understood. In limited clinical studies, BBB permeability measurements after TBI are diverse. Increased CSF to serum albumin quotients suggests acute BBB dysfunction at 12 h–76 h [44–47], with some cases remaining elevated up to 22 days post-injury compared to control patients [47, 48]. Furthermore, higher resolution imaging demonstrates BBB disruption persisting for months/years at the site of injury following TBI [49, 50]. Consistent with clinical studies, conflicting data on the time course of BBB disruption is also reflected in animal models. Crucially, the effect of experimental TBI on brain permeability is likely to differ according to species, injury model, severity of impact and post-injury interval time. Indeed, rat TBI models (using variants of open or closed head injuries, mostly report BBB permeability at acute time points (30 min-24 h) [51–56], with few extending out to 3-days post injury [57]. Similarly, mouse models of TBI focus on BBB assessment acutely (6 h-3 days), with many comparing pharmacological treatments at a single time point, most often 24 h post injury [58–63]. These studies all share common limitations, as most do not verify the overall time-course of BBB permeability specific to their TBI model. It is important to note that BBB permeability is more complex than simply an open or closed state. It’s dynamic structure exists in a continuum after TBI, ranging from primary injury rupture to various states of permeability due to secondary injury-induced biochemical alterations, which can change functional profile over time [64]. As such we sought to comprehensively characterize the time-course of BBB permeability following TBI. To the best of our knowledge, this study is the first to report the overall time course (24 h-21 days) of BBB permeability, including BBB rupture, stabilization and closure, in a CCI mouse model of TBI. Using serum albumin detection and MRI visualization, our findings demonstrate dynamically increased BBB permeability at the ipsilateral lesion for the first 3 days post-TBI, before resolution 7–10 days post-injury. We also used pharmacological intervention to show that CypA is a functional target, with CypA inhibition shifting the BBB repair curve towards a faster resolution.

The APOE gene encodes for the 34 kDa protein apoE, which serves as a major lipid transport molecule in the CNS [65]. Transcriptomic analysis of TBI-induced alterations in genomic profiles, has revealed elevated apoE signatures after injury. Moreover, translational integration analysis of this apoE response demonstrates a significant profile overlap between experimental TBI and human GWAS neurodegeneration studies [66], enhancing the notion that apoE plays a pertinent role after brain injury. Indeed, apoE^−/−^ mice display progressive, age-dependent BBB leakage [10, 11] which appears to be exacerbated with injury [10], suggesting that a lack of apoE may increase BBB dysfunction and edema following brain trauma. Despite this, time-course analysis of how apoE may mediate BBB integrity after TBI has largely been unexplored. In the present study, we report what appears to be a biphasic, inverse relationship between apoE (mRNA and protein expression) and BBB dysfunction, such that BBB permeability decreases as apoE levels increase over time post-TBI. This biphasic pattern of a reduction in PBS-soluble apoE followed by an increase is consistent with our previous finding with diethylamine-soluble apoE following experimental TBI [8], and with clinical reports documenting a 70% reduction in CSF derived apoE acutely following severe TBI [7]. The mechanisms by which apoE is degraded or removed from the soluble fraction after TBI remain unclear. *Apoe* expression is controlled by an LXR/RXR responsive element, and we have shown that levels of the CYP46 enzyme responsible for production of the endogenous LXR agonist 24S-hydroxycholesterol are increased after TBI [67]. Furthermore, both apoE and another LXR-responsive protein, ABCA1, are increased at 3 and 7 days after experimental TBI in mice and rats [67, 68]. While we have not directly measured the lipidation status of apoE produced after TBI, all of the elements are in place to rapidly produce and lipidated apoE in the sub-acute phase post-TBI.

ApoE is primarily produced in astrocytes [69] and with robust gliosis in our model of TBI, our FISH and protein imaging show that astrocytes remain the primary site of *apoe* production after injury. However, we also detect *apoe* mRNA signal from pericytes. Isolated brain pericytes have previously been shown to be capable of producing apoE [70, 71], and here we provide evidence that this also happens in vivo. Brain pericytes play a crucial role in maintaining and regulating the integrity of the BBB, through their ability to modulate endothelial tight junction expression and transcytosis of blood-derived macromolecules [31, 32]. ApoE regulates BBB integrity within the NVU by signaling through LRP1 on pericytes, suppressing downstream CypA-MMP-9 responses that degrade tight junction proteins in the vascular endothelium [9]. Critically, this pathway is controlled in an isoform-specific manner, with MMP-9 responses not suppressed by apoE4, resulting in enhanced BBB dysfunction [9]. We have also previously shown that APOE4 mice have less soluble apoE than APOE3 mice up to 7 days post-TBI [8], which could interfere with the ability of apoE4 to suppress MMP-9 production in microvessels after TBI.

In the present study, we sought to comprehensively characterize the TBI induced NVU response in C56Bl/6 mice, before moving into the clinically relevant APOE3 and APOE4 mouse model. Unlike previous studies that employ whole brain methodologies to analyze BBB properties, here we utilized isolated microvessels to analyze specific NVU cell types. This approach has been previously demonstrated to provide an isolation containing with enriched pericyte and endothelial expression, with minimal transcripts derived from astrocytic end feet suggesting glial cells have negligible contributions to the responses observed in microvessel preparations [72]. In the current study, ipsilateral microvessels from C57Bl/6 mice exhibit increased MMP-9 expression up to 3 days post-injury, even in the presence of concurrent pericyte loss as demonstrated by reduced PDGFRβ expression. Given pericytes are key producers of MMP-9 at the NVU [73], this data highlights that despite TBI induced pericyte loss, those that do remain at the NVU following injury are still capable of robust MMP-9 production. Consequentially, this response appears to contribute to endothelial dysfunction within the NVU, with reduced expression of key tight junction elements Zonula Occludens-1 and Occludin up to 3 days post-TBI. Significantly these NVU injury responses are attenuated in the presence of microvessel specific apoE, with the time dependent increase in apoE (3–7 days) corresponding with reduced MMP-9 and restored expression of tight junction proteins, indicative of BBB stabilization.

Recent research indicates that apoE is not having a direct signaling effect on endothelial cells, as targeted deletion of LRP1 from brain endothelial cells does not affect BBB integrity [74]. Rather, we believe that microvessel-derived apoE exerts its effects through LRP1 mediated inhibition of MMP-9 responses in remaining pericytes after injury, subsequently suppressing a stimulus which contributes to tight junction degradation following TBI. Supporting this, our experiments using the CypA antagonist CsA, demonstrate reduced microvessel specific MMP-9 levels with no change in pericyte expression, resulting in greater stabilization of BBB integrity.

The role of APOE polymorphisms regulating BBB function is rapidly becoming an emerging field of interest in the development of genotype-specific therapies. In non-injury paradigms, APOE4 but not APOE3 mice display dysfunctional BBB properties that contributes to exacerbated secondary injury and cognitive decline [9, 75]. Furthermore, individuals carrying the APOE4 gene in Alzheimer’s Disease display accelerated pericyte degeneration [22]. A recent experimental TBI paper also found that whole cortex homogenates from APOE4 mice have reduced tight protein compared to APOE3 mice at 7 days post-injury, alongside increased MMP-9 activity [76]. Our results show that at 10 days post-injury, closure of the BBB has occurred in APOE3 mice. In contrast, APOE4 mice do not display significant reductions in BBB permeability at 10 days compared to 3 days post-injury when BBB leakage is evident. This demonstrates that the E4 isoform impairs the closure of the BBB following TBI. Mechanistically, our data suggests differential roles of APOE polymorphisms in regulating multiple pericyte-derived responses, which may cumulatively combine to be key determinants of BBB permeability after TBI. We observe a time dependent loss of PDGFRβ expression acutely after TBI. This is pertinent given previous studies evidencing its critical role, with PDGFRβ deficiency resulting in pericyte loss, increased BBB permeability and enhanced secondary injury processes [31, 32, 77]. Both APOE3 and APOE4 mice display reduced PDGFRβ expression up to 3 days post-TBI, consistent with a recent study utilizing similar trauma parameters showing rapid pericyte loss (PDGFRβ staining) in wildtype mice, starting at 6 h and lasting for 3 days post-injury [78]. However, in comparison to C57Bl/6 and APOE3 mice that display recovery of PDGFRβ expression at 7 days, APOE4 mice exhibit significantly prolonged reductions in PDGFRβ. This may be indicative of either reduced pericyte proliferation or delayed pericyte recruitment and/or migration back to the site of the pericontusional lesion following injury, as has been previously reported [79].

Given pericytes are considered a key source of MMP-9 production/release at the NVU following injury stimuli [73, 80], our results demonstrating augmented and sustained MMP-9 expression in APOE4 microvessels suggests that altered signaling in remaining pericytes contributes to the isoform dependent regulation of BBB integrity following TBI. Consistent with our data, not only is MMP-9 rapidly upregulated in human TBI patients [81, 82], clinical evidence suggests that this may be exacerbated in the presence of the APOE4 allele. Cognitively normal human apoE4 carriers exhibit increased CSF expression of MMP-9 correlating with BBB dysfunction, whilst post-mortem AD brains display elevated MMP-9 in cortical pericytes, in an isoform dependent manner (APOE4 > APOE3) [22]. At the vascular level, there was no difference between genotypes in the total density of regenerating/surviving microvessels in the pericontusional area after TBI, as identified by CD31 staining. Tomato-lectin is widely used to stain blood vessels through its binding to *N*-acetylglucosamine oligomers, trimers and tetramers expressed in the endothelium. However, *N*-acetylglucosamine is also expressed in glial cells [83], including those that are involved in the formation of the glial scar after TBI [84, 85]. As a result lectin stains glial cells [86, 87], and in our experiments we found wide-spread lectin staining in the glial scar and surrounding cortex that made it impossible to quantify lectin-positive blood vessels in this area – even though these blood vessels were still clearly visible. What we do find is that APOE polymorphisms differentially regulate tight junction mRNA recovery at the NVU after TBI. APOE4 mice displayed reduced levels of Zonula Occludens-1, Occludin and Claudin-5 compared to APOE3 post-injury, enhancing the notion that APOE4 significantly prolongs BBB dysfunction after TBI. Importantly we have shown that inhibition of the CypA-MMP-9 pathway can enhance BBB repair after TBI. As this step is downstream of the apoE-LRP interaction, this remains a plausible target for reversing the detrimental effect of apoE4 on BBB repair after TBI.

## Conclusion

In conclusion, we have identified a role for apoE in contributing to BBB permeability, stabilization and closure after TBI. Specifically, the presence of APOE4 allele prolongs a variety of deleterious cellular responses that results in the overall delay of spontaneous BBB closure after injury. This data may help explain the mechanisms underlying the unfavorable recoveries of APOE4 carriers following TBI. Future studies investigating genotype-specific therapies targeting the BBB may prove beneficial to improving outcomes after TBI.

## Abbreviations

APOE: apolipoprotein-E
BBB: Blood-brain barrier
CCI: controlled cortical impact
CNS: Central nervous system
CsA: Cyclosporine A
CSF: Cerebrospinal fluid
CypA: Cyclophilin A
EB: Evans blue
GWAS: Genome wide association study
LRP1: Low density lipoprotein receptor-related protein-1
MMP: Matrix metalloproteinase
MRI: Magnetic resonance imaging
NVU: Neurovascular Unit
RT-QPCR: Real time quantitative polymerase chain reaction
TBI: Traumatic Brain Injury
